# Nonalcoholic Fatty Liver Disease and Hepatocellular Carcinoma

**DOI:** 10.1155/2014/106247

**Published:** 2014-03-11

**Authors:** Luciana Kikuchi, Cláudia P. Oliveira, Flair J. Carrilho

**Affiliations:** São Paulo Clínicas Liver Cancer Group, Hospital das Clínicas, Instituto do Câncer do Estado de São Paulo, Department of Gastroenterology, University of São Paulo School of Medicine, Avenida Dr. Enéas de Carvalho Aguiar No. 255, Instituto Central No. 9159, 05403-000 Sao Paulo, SP, Brazil

## Abstract

Hepatocellular carcinoma (HCC) incidence is increasing worldwide in recent years. Most HCC cases develop in the presence of advanced chronic liver disease related to chronic hepatitis C virus (HCV) infection, chronic hepatitis B (HBV) infection, and alcohol abuse. Approximately 15–50% of HCC cases are classified as idiopathic, suggesting that other risk factors are responsible for its rising incidence. Recent studies suggest that nonalcoholic fatty liver disease (NAFLD) can be associated with these “idiopathic” cases. NAFLD progresses slowly and can develop into liver cirrhosis, liver failure, and HCC. In the last few years, NAFLD has received more attention because of its high prevalence worldwide.

## 1. Introduction

Hepatocellular carcinoma (HCC) is considered the 5th most common cancer in the world and is responsible for 5% of all malignant tumors in humans [1]. In recent years, a rising incidence has been observed in many countries including America, Europe, and Asia [2, 3]. Most HCC cases develop in the presence of advanced chronic liver disease related to chronic hepatitis C virus (HCV) infection, chronic hepatitis B (HBV) infection, and alcohol abuse. Approximately 15–50% of HCC cases are classified as idiopathic, suggesting that other risk factors are responsible for its rising incidence [3]. Cryptogenic cirrhosis (CC) is observed in 5–30% of patients with advanced liver disease. Recent studies suggest that nonalcoholic fatty liver disease (NAFLD) can be associated with these “idiopathic” cases [4]. NAFLD progresses slowly and can develop into liver cirrhosis and liver failure. In the last few years, NAFLD has received more attention because of its high prevalence worldwide [5]. The theory that HCC is part of the natural history of NAFLD comes from 4 research fields: (1) retrospective studies that demonstrated HCC, which is developed from CC, related to NAFLD risk factors; (2) case reports; (3) prospective studies that evaluated late complications from NAFLD patients; and (4) metabolic risk factors, hepatocarcinogenesis, and animal models.

### 1.1. Retrospective Studies: Hepatocellular Carcinoma Was Associated with Cryptogenic Cirrhosis

In a retrospective case-control study, the prevalence of CC in 641 patients with HCC was 6.9%. Risk factors for NAFLD, mainly obesity and diabetes, were more common in patients with CC when compared to patients with chronic hepatitis and alcoholic liver cirrhosis. The prevalence of obesity before the onset of cirrhosis was 41% in CC compared to 16% in the control group (*P* = 0.008). Diabetes mellitus was twice as prevalent in CC as the control group (50% versus 20%, *P* = 0.0034). Previous dyslipidemia, mainly hypertriglyceridemia, was observed in 22% and 5% of patients in the CC and control group, respectively. The majority of patients were male (74% and 72%, resp.) in both groups, but patients in the CC group were older (69 versus 64 years of age, *P* < 0.001). Tumor findings were similar in both groups; most patients presented with a solitary lesion measuring less than 30 mm [4].

In a cohort of 105 HCC patients, CC was the second etiology (29%), only after HCV (51%). Compared to others etiologies, the majority of CC patients were female (60% versus 28%, *P* = 0.001), obese (58% versus 25%, *P* = 0.02), diabetic (47% versus 8%, *P* = 0.006), and hypertriglyceridemic (16% versus 2%, *P* = 0.001). In 20% of CC cases, liver biopsies showed steatosis, lobular inflammation, and ballooning degeneration, but steatosis was severe (more than 30% of hepatocytes) in only half the patients. Fifty percent of patients had a histologic diagnosis or clinical suspicion of NAFLD. In this cohort, 13% of HCC cases were considered related to NAFLD. In this group, tumors from CC patients were larger and the chance of receiving a potentially curative therapy was limited, which can be explained by less frequent HCC surveillance in CC patients [6].

Between January 2010 and December 2012, 42 patients with HCC related to either NAFLD or CC were retrieved retrospectively from 2 centers in Brazil (Instituto do Câncer do Estado de São Paulo and Universidade Federal do Rio Grande do Sul). This included patients from an observation study of HCC in NAFLD of the FLIP consortium. The median age of the patients was 66.5 years (range of 25–80 years) and male gender predominated (*n* = 26; 62%). Thirty (71%) of NAFLD-related cases and 12 (29%) CC cases were collected over 24 months. There were 4 patients without evidence of cirrhosis according to liver biopsy and/or clinical evaluation. In the NAFLD group, most patients (81%) presented with metabolic syndrome risk factors, such as obesity, diabetes, arterial hypertension, or dyslipidemia. HCC was diagnosed in a screening program in 55% of the 42 patients (there was one noncirrhotic patient). HCC was diagnosed based on the noninvasive diagnostic criteria of the American Association for the Study of Liver Diseases (AASLD) in 24 patients (57%) and the diagnosis was confirmed by histology in 18 patients (43%, unpublished results).

### 1.2. Case Reports of HCC in Cirrhosis Related to NAFLD

Clinical and histological characteristics of 16 HCC cases associated with NAFLD, reported until 2007, were described in a review study from Bugianesi [7] (Table 1). Most patients were male, and the age at diagnosis varied from 56 to 74 years (average of 66 years). One or more risk factors for metabolic syndrome were present at the NAFLD diagnosis, mainly obesity and diabetes. In half the cases, HCC was diagnosed at the first medical visit. In the remaining cases, the interval between liver disease diagnosis and HCC varied from 6 months to 10 years. HCC was multifocal or larger than 30 mm in 50% of cases, but well differentiated in most cases. Seven patients were eligible for hepatic resection, and local ablative therapy was used in others. In most cases, the serum alpha-fetoprotein was normal at HCC diagnosis.

In 2009, our group reported 7 cases of HCC in patients with NAFLD confirmed by histology. Four patients were male and the median age was 63 years. Obesity and diabetes were observed in 57% of patients and one patient was noncirrhotic. Among cirrhotic patients, most of them (71%) had a Child-Pugh score of A. Four patients presented with multifocal HCC and the tumor size varied from 10 to 52 mm with 57% of cases having a tumor smaller than 30 mm. All patients had alpha-fetoprotein levels less than 100 ng/mL at diagnosis. For HCC therapy, liver resection was performed in 2 patients, transarterial chemoembolization in 3 patients, percutaneous ethanol injection in 2 patients, and liver transplant in 1 patient [8].

### 1.3. Prospective Studies of Nonalcoholic Steatohepatitis (NASH) and HCC

The natural history of NASH has been evaluated in cohort studies and liver biopsy sequential studies to analyze the clinical outcome and progression to cirrhosis and HCC. The main limitations of these studies are the reduced number of patients and short follow-up periods. There are few studies that correlate HCC development in patients with NAFLD, probably due to the low rate of fibrosis progression [9].

A study that evaluated 420 patients with NAFLD identified cirrhosis in 5% after a follow-up period of 7 years. Only 3% of patients progressed to cirrhosis, including 2 cases of HCC. Survival was shorter than in the general population and liver disease was the third cause of death in this group of patients (after cancer and cardiovascular disease). Only 2 out of 420 patients developed HCC (0.5%), but this rate was 10% in patients with liver cirrhosis (2 out of 21 patients). Results from this study confirmed the bad prognosis in patients with NAFLD-related cirrhosis; 33% of patients died from complications of liver disease, suggesting that surveillance may be helpful in this subgroup [10]. We anticipate that prospective studies about the natural history of NAFLD with longer follow-up periods and larger numbers of patients will determine the real incidence of HCC in patients with NAFLD-related cirrhosis.

Risk factors for HCC were evaluated in another prospective study that followed 137 Japanese patients with NAFLD since 1990. The median age was 70 years and around 88% of patients with HCC had advanced liver fibrosis. Other risk factors for HCC were lower aminotransferases levels and histological activity. The cumulative incidence of HCC was 7% in 5 years and it was the main cause of death. Surveillance for early detection of HCC in patients with NAFLD and advanced fibrosis was highly recommended in this study [11].

One study compared the risk of developing HCC in NASH- to HCV-cirrhotic patients. Yearly cumulative incidence of HCC was found to be 2.6% in patients with NASH-cirrhosis, compared with 4.0% in patients with HCV-cirrhosis (*P* = 0.09) [12]. Another study compared the risk of developing HCC in NASH-cirrhotic to alcohol liver disease patients, and the development rates of HCC of these 2 conditions were quite similar [13].

HCC can be considered a rare complication in patients with NAFLD, but it should not be underestimated. First, HCC is a common complication after the establishment of cirrhosis, with an approximately 7 to 21% prevalence, mainly in obese and overweight patients, and an incidence of 10% during follow-up (7 years) [10]. Second, the HCC diagnosis is generally performed during the first visit of the patient and the tumor is often multifocal with large dimensions that limit the available treatments [6]. Third, patients are older and have more comorbidities at the time of HCC diagnosis, which may reduce the applicability of potentially curative therapies, such as liver transplantation [11]. These findings justify a surveillance program mainly after the establishment of cirrhosis.

### 1.4. Metabolic Risk Factors, Hepatocarcinogenesis, and Animal Models in NAFLD

The mechanisms of hepatocarcinogenesis in NAFLD patients are related to cirrhosis and underlying disease (e.g., carcinogenic potential of steatosis and metabolic dysregulation). In obese and diabetic patients, HCC development can be attributed to the presence of NAFLD. Evidence suggests that adiposity and diabetes can increase the incidence, death rate, or both in a variety of malignant neoplasms in human beings [14].

## 2. Metabolic Syndrome and the Risk of HCC

### 2.1. Obesity and HCC

Obesity is recognized as an important risk factor for carcinogenesis in many malignant neoplasms. A National Cancer Institute study followed 900,000 adults from 1982 to 1998 and registered more than 57,000 cancer-related deaths [14]. These data indicate that weight is associated with higher cancer mortality. In patients with a BMI ≥35 kg/m^2^, mortality rates were 52% higher in men and 62% higher in women. A linear positive correlation was observed in Hodgkin lymphoma, multiple myeloma, and colorectal, liver, breast, gallbladder, pancreas, uterus, cervix, and kidney cancers. Among men, liver cancer showed the highest relative risk increase.

Obesity also represents a risk factor for HCC in patients with cirrhosis from other etiologies. In liver transplant patients in the USA, the HCC incidence was a little higher in obese patients (4% versus 3%, *P* = 0.013). Multivariate analysis identified obesity as an independent risk factor for HCC in cryptogenic and alcoholic cirrhosis but not in HCV, HBV, autoimmune hepatitis, and primary biliary cirrhosis [15]. This finding suggests that HCC in obese patients may be related to two factors: NAFLD and the carcinogenic effect of obesity* per se*.

### 2.2. Diabetes and HCC

Epidemiologic studies demonstrated an increased risk for HCC in patients with diabetes mellitus type 2 (DM2). In cohort studies, patients with DM2 presented with a 3-fold increased risk of developing HCC and, in the presence of hepatitis, cirrhosis, and alcohol abuse, this risk increased 4-fold [16]. In a case-control study, HCV and DM2 increased the HCC risk 37-fold (*P* < 0.0001), suggesting a synergic effect [17].

The incidence of HCC among patients with (*n* = 173,643) or without DM2 (*n* = 650,620), excluding all patients that had liver disease before or in the first year of follow-up, was analyzed in a large longitudinal study. During the observational period of 10 to 15 years, the incidence of HCC increased more than 2-fold among diabetics (incidence rate = 2.39 versus 0.87/100,000 persons/year), and was higher in patients with longer follow-up times. There was evidence for a causal relation based on DM2 preceding the development of HCC and the DM2 time exposure [18].

The absolute risk for HCC development in patients with DM2 and obesity can be considered low; however, the pandemic of these two diseases can transform this small number into a great number of HCC cases. In fact, almost 35% of the adult population in the USA and a great proportion of the worldwide population are overweight or obese, including children [19, 20], and this is associated with a higher prevalence of DM2. In a period of one year, the DM2 prevalence increased from 7.3% to 7.9% in the USA general population [21]. In the next decades, HCC cases related to obesity and DM2 are projected to increase, while viral etiology cases will tend to decrease.

## 3. Hepatocarcinogenesis in NAFLD

Most HCC cases are diagnosed in patients with cirrhosis after long term follow-up. It is not clear, however, if the neoplastic process begins after the establishment of cirrhosis or in earlier stages of liver disease. Steatosis* per se* and the physiopathological mechanisms of NAFLD have carcinogenic potential. During the carcinogenesis, epithelial hyperplasia and dysplasia generally precede malignant tumors by many years [22]. In human beings, HCC has rarely been identified in a liver with only steatosis. In ob/ob rats (insulin-resistant and obese), HCC develops in the absence of cirrhosis [23]. In ob/ob rats, the proliferation of hepatocytes is increased compared to apoptosis, suggesting that this misbalance promotes an increase in liver mass [24]. The absence of inflammation or fibrosis suggests that cell survival is promoted by insulin resistance in hepatocarcinogenesis.

**Figure 1:**
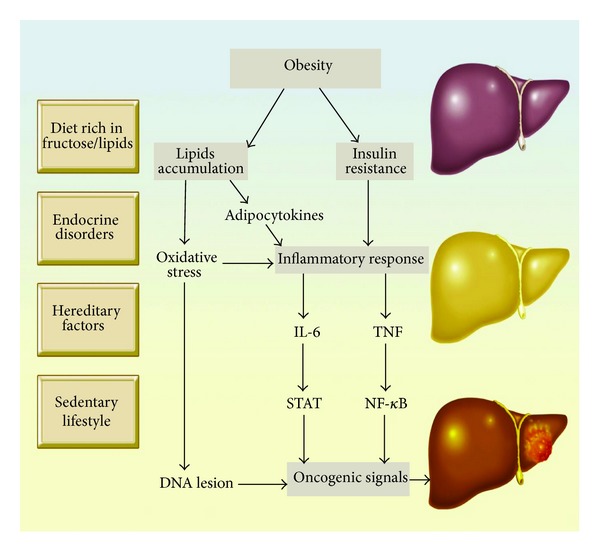
Mechanisms of carcinogenesis in NAFLD adapted from: Sun & Karin, Journal of Hepatology, 2011; Toffanin, Friedman, Llovet, Cancer Cell, 2010.

Most molecular events that lead to HCC need better clarification, but the main steps to cancer development (initiation, promotion, and progression) present a clear correlation with the NAFLD physiopathology [25]. Obesity is related to insulin resistance and augmentation of insulin-like growth factors, which act as a mitogen to stimulate cellular growth [26] (Figure 1). In addition, obesity is associated with hyperestrogenemia, which is also implicated in the proliferation of hepatocytes [27].

Proliferation of oval cells (progenitor cells of hepatocytes, which have been implicated as the origin of many liver tumors) has been observed in many patients with NAFLD in experimental studies [28]. Increased production of reactive oxygen specimens and DNA oxidative injury may also contribute to HCC development [29, 30]. Oxidative stress can contribute to mutations in regulatory genes, including tumor suppressors such as p53 and PTEN (phosphatase tensin homolog) [31]. Also, elevated production of ROS can increase fatty acids in the endoplasmic reticulum or peroxisomes and can modulate PPAR-alpha by intrahepatic lipids. Sustained activation of PPAR-alpha induces HCC in AOX-absent, PPAR-alpha, and PPAR/alpha/AOX mice and rats, probably through transcriptional activation of regulatory genes of PPAR-alpha and ROS generation.

Recently, our group published a tissue microarray study that demonstrated, in all spectrums of NAFLD, that Survivin, an antiapoptotic protein, was expressed differently in NASH-HCC related tissues compared with HCV-HCC related tissues [32]. Also, in another tissue microarray study, we identified that mTOR was differently expressed in NAFLD cirrhosis compared with other causes of cirrhosis [33]. These findings suggest that carcinogenesis-related NAFLD can follow different molecular pathways to fibrosis-cirrhosis HCC.

Several small animal models of nonviral HCC have been characterized and demonstrated that carcinogenesis in NAFLD has peculiar aspects. The liver-specific Pten deficient mouse is a transgenic mouse that develops NASH, adenomas, and HCC and is bred by mating Ptenflox/flox mice with Alb-Cre transgenic mice. These animals develop steatohepatitis associated with cancer cell expression of PPAR*γ*. HCC was seen in 83% of animals that were 74–78 weeks old [34].

Fatty change, cirrhosis, and HCC have also been described in Sprague Dawley and Fisher rats exposed to diethylnitrosamine, administered either weekly by intraperitoneal injection for 3 weeks or daily in the drinking water (110 mg/L) for 10 weeks. Following exposure, 94% (68 of 72 animals) developed HCC by 37 weeks [35]. In 2008, our group published a rodent model that replicates many features of NASH including steatohepatitis, ballooning, fibrosis, cirrhosis, and HCC. Oval cell proliferation was evident and the presence of anti-CK 19 positivity in the cancer suggested that the malignancy is originated in oval cells. This model developed HCC in 16 weeks [36]. Recently, Hoshida et al. published a review describing some animal models of HCC, including NAFLD-HCC models [37].

## 4. Final Considerations

Studies about HCC in NAFLD are scarce and are mostly from retrospective studies and case series. The long and indolent progression of the disease limits prospective studies. HCC seems to be a rare but disturbing complication of NAFLD that is related to the higher incidence of metabolic syndrome. Cirrhotic patients with NAFLD show important risk factors for HCC development and have a worse prognosis because they are older and present with other comorbidities. The role of cirrhosis, steatosis, and metabolic derangement in hepatocarcinogenesis needs to be elucidated. A better understanding of genetic and metabolic determinants of hepatocyte growth and differentiation can lead to the development of new pharmacological therapies. The main efforts must be directed to NAFLD prevention through promotion of healthy measures.

## Figures and Tables

**Table 1:** Case reports of HCC associated with NAFLD [7].

Case number	Age (years)	Sex	Comorbidity	Interval between liver disease and HCC (years)	Number/size (cm) HCC	Liver histology	Treatment	Survival
1	52	F	DM	4	Mult/—	Cirrhosis	Resection	Dead
2	62	M	DM, Ob	4	1/3	Cirrhosis	PEI	Dead
3	72	F	DM	10	3/1.4	Cirrhosis	NR	NR
4	67	F	DM	0	1/2.6	Fibrosis	Resection	NR
5	66	F	DM	2.5	1/1.5	Cirrhosis	Resection	Recurrence
6	68	F	NR	2	1/2	Cirrhosis	TAE	Alive
7	69	F	DM, Ob	0.5	1/2.5	Cirrhosis	TAI	Recurrence
8	72	M	Ob	0	1/3	Cirrhosis	TAE, PEI	Recurrence
9	63	M	DLP, Ob	0	1/2	Cirrhosis	Resection	Alive
10	56	M	DM	0	Mult/6	Cirrhosis	TAE	Dead
11	76	M	DM, Ob	10	1/1.9	Cirrhosis	RFA	Alive
12	74	M	DM, Ob	0	1/4	Fibrosis	Resection	NR
13	64	M	DM, Ob	0	1/—	Steatosis	TAE, resection	Alive
14	67	F	Res. Ins	2	2/1.5	Cirrhosis	TAE	Dead
15	64	M	Ob, DLP	0	Mult/13	Fibrosis	NR	Dead
16	70	M	DM, Ob	0	1/4.5	Cirrhosis	TAE, resection	NR

M: male, F: female, DM: diabetes mellitus, Ob: obese, DLP: dyslipidemia, Mult: multinodular, PEI: percutaneous ethanol injection, TAE: transarterial embolization, TAI: transarterial chemotherapy infusion, RFA: radiofrequency ablation, and NR: not reported.
